# Supplementary material for: Identification of Immune Infiltration and the Potential Biomarkers in Diabetic Peripheral Neuropathy through Bioinformatics and Machine Learning Methods
Source: Biomolecules. 2022 Dec 26;13(1):39. doi: 10.3390/biom13010039 (PMC9855866; doi:10.3390/biom13010039)
Supplement: Supplementary file 1 [file biomolecules-13-00039-s001.zip › Supplementary Materials.pdf]

Supplementary Materials

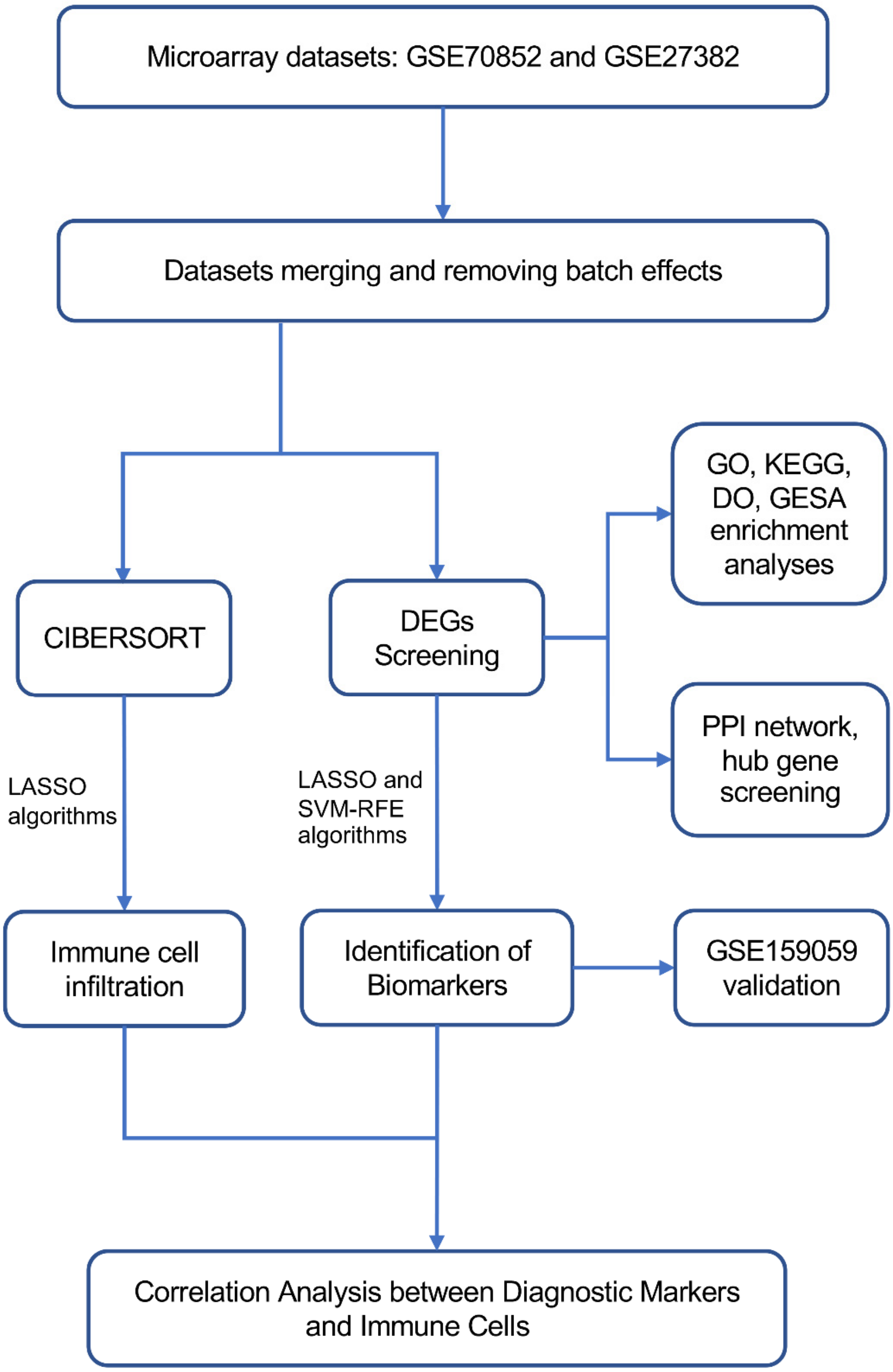

Figure S1. The flow diagram of this study.

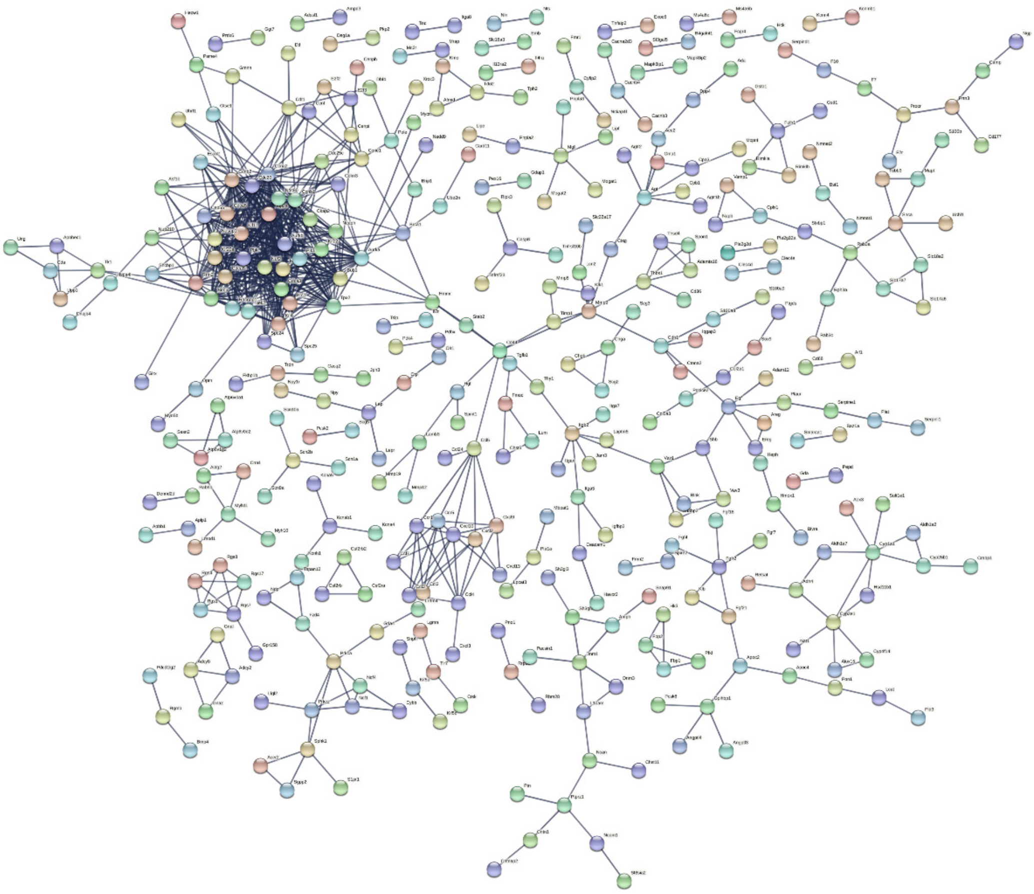

Figure S2. PPI network constructed using STRING database with interactions combined score > 0.9 being used for network construction

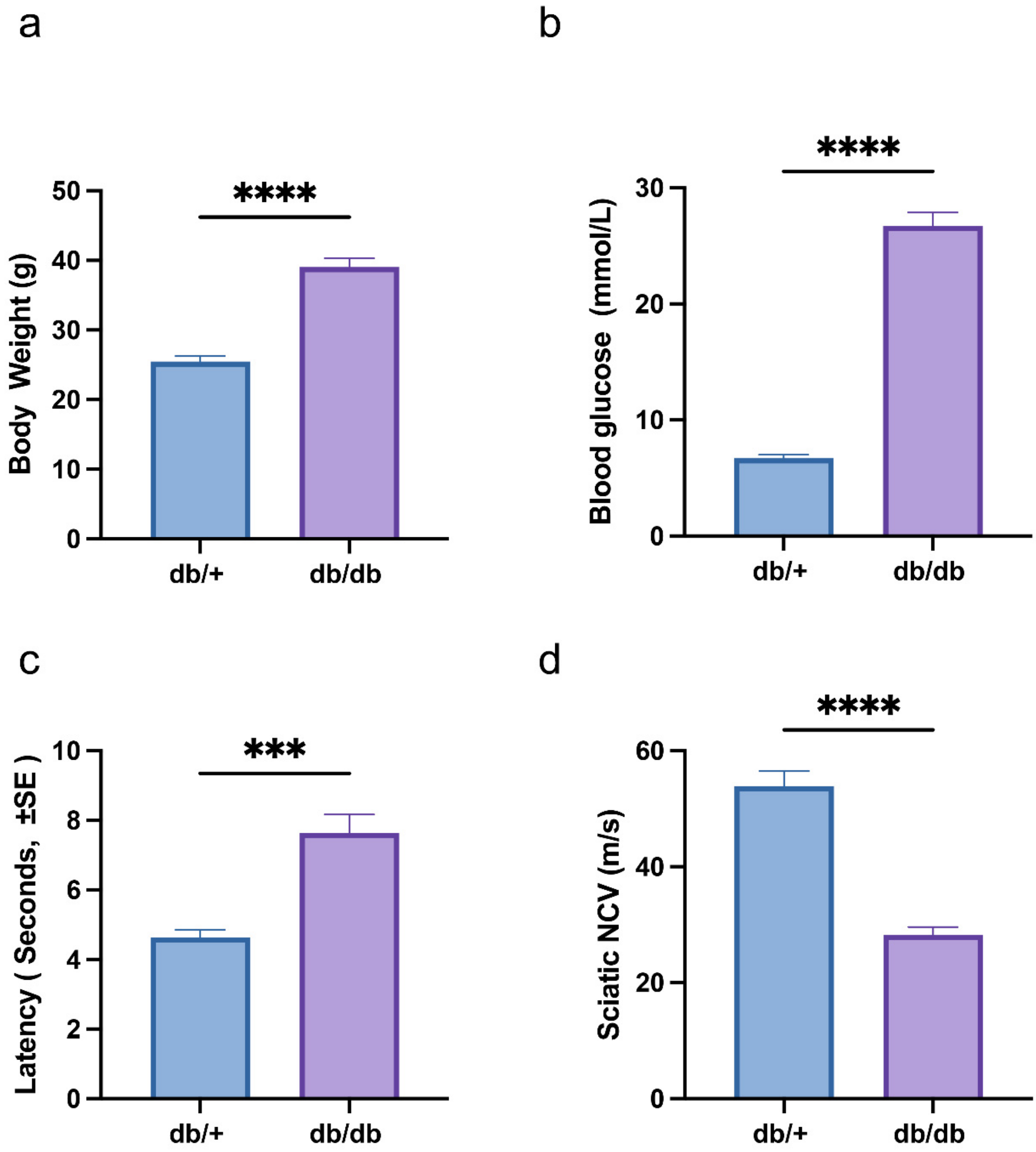

Figure S3. The metabolic phenotyping and neuropathy phenotyping in mice. **a** Body weight, **b** blood glucose, neurological function measured by **c** Hind paw withdrawal latency and **d** sciatic motor nerve conduction velocity (NCV), *, $p<0.05$, **, $p<0.01$, ***, $p<0.001$, ****, $p<0.0001$.

Table S1. Primers used for qRT-RCR in this study.

UBD	Forward	CCAATGGCGGTTAATGACCTT
	Reverse	TTTCGATGGGGCTTGAGGATT
UCP1	Forward	AGGCTTCCAGTACCATTAGGT
	Reverse	CTGAGTGAGGCAAAGCTGATTT
LTBP2	Forward	CTGCCCCAGTGGTCAAGGTTA
	Reverse	GCACTATGTTGAGGCATCGGC
CCL2	Forward	TTAAAAACCTGGATCGGAACCAA
	Reverse	GCATTAGCTTCAGATTTACGGGT
S100A8	Forward	AAATCACCATGCCCTCTACAAG
	Reverse	CCCACTTTTATCACCATCGCAA
HSPB7	Forward	GAGCATGTTTTCAGACGACTTTG
	Reverse	CCGAGGGTCTTGATGTTTCCTT
GPNMB	Forward	TGCCAAGCGATTTCGTGATGT
	Reverse	GCCACGTAATTGGTTGTGCTC
MMP9	Forward	AACCTCCAACCTCACGGACAC
	Reverse	GGTCCCACTTGAGGCCTTTGA
PON1	Forward	TTGGCATTTCCCTTGATGGCA
	Reverse	ATTCGAAGCACCTCTGAGCCG
CYP2F2	Forward	AAAGGGGAGGAGTTCAGTGGC
	Reverse	GGATCTTCCAGCGCTCTCCAT
CDH1	Forward	ACTGCCAACTGGCTGGAGATT
	Reverse	GTGGGTTCCTCTGGCAGAACT
TUBB3	Forward	GCGGCAACTATGTAGGGGACT
	Reverse	ATAGTGCCCTTTGGCCCAGTT
MYT1L	Forward	TGGTCACGTCAGTGGCAAATA
	Reverse	TGCAAATGGTTTTCGCTTGGG
CAANB4	Forward	TACCTGCATGGAGTTGAAGACT
	Reverse	TTCGCTCTCTCAAGCTGGATA
MGL2	Forward	TTAGCCAATGTGCTTAGCTGG
	Reverse	GGCCTCCAATTCTTGAAACCT
β-actin	Forward	GCCTCCCAATCTCTCCATGCT
	Reverse	AGCTGGGCAGAGAAAGGGATG
